# Unilateral Pulmonary Fibrosis Due to Absence of Right Pulmonary Artery

**DOI:** 10.7759/cureus.5161

**Published:** 2019-07-17

**Authors:** Hasan Sumdani, Zanab Shahbuddin, Nabeel Farhataziz, John M Barkley

**Affiliations:** 1 Neurosurgery, Texas A&M College of Medicine, Round Rock, USA; 2 Emergency Medicine, University of North Texas Health Science Center, Fort Worth, USA; 3 Neuroradiology, Austin Radiological Association, Austin, USA; 4 Radiology, Austin Radiological Association, Austin, USA

**Keywords:** pulmonary fibrosis, lung disease, pulmonary artery, vascular disease, variant anatomy, reactive oxygen species, oxidative stress, congenital

## Abstract

Pulmonary fibrosis is typically a bilateral, progressive interstitial lung disease that is often idiopathic but can be associated with risk factors such as advanced age, environmental exposure, and drug toxicity. The pathophysiology is incompletely understood but involves transforming growth factor. The treatment choices for idiopathic disease include medical therapy that manipulates epigenetic pathways and lung transplantation. Here we present a 30-year-old female with no identifiable risk factors who developed unilateral pulmonary fibrosis. Clinical investigation eventually revealed a congenitally absent right pulmonary artery which was presumed to cause her unilateral disease. In contrast to idiopathic pulmonary fibrosis, treatment options for pulmonary fibrosis due to unilateral absence of a pulmonary artery include ipsilateral pulmonary vasculature embolization and/or pneumonectomy if disease is unmanageable without therapy.

## Introduction

Pulmonary fibrosis (PF) is a progressive, chronic, interstitial lung disease that is a common end result of many triggering processes including smoking, environmental exposure, drug toxicity (e.g., amiodarone, bleomycin), and radiation treatment/exposure, however, the cause is often idiopathic [1,2]. The disease is almost always bilateral since both lungs usually have the same exposure to environmental triggers. Despite the increase in our understanding of PF, its incidence continues to rise. The incidence of PF follows certain patterns such as increasing with increasing age and preferentially affecting males rather than females in the United States [3]. Risk increases with genetic predispositions and respiratory or other comorbidities.

The onset of PF is insidious, and the main symptomatology includes progressive shortness of breath with steady decline in exercise tolerance and cough [3]. Though idiopathic in many cases, PF has been seen to be associated with conditions including gastroesophageal reflux disease (GERD) and diabetes mellitus, and incidence of other respiratory conditions is increased in patients with PF when compared to controls. These conditions include pulmonary hypertension, emphysema, coagulopathy (pulmonary embolism), infection, and cancer [4].

The specific mechanism of PF manifestation varies with the etiologic agents responsible, but their pathophysiologies converge to increasing the activity of transforming growth factor beta (TGF-β) which provokes replacement of epithelial gas exchange surfaces with connective tissues that do not provide any gaseous exchange functionality [5,6].

In the setting of PF with ipsilateral absence of the pulmonary artery, the mechanism is thought to be due to parenchymal hypoperfusion, however, other mechanisms are also speculated and include reactive oxygen species (ROS) [7,8].

The best therapy for idiopathic PF is lung transplant, but this is unavailable to the majority of patients. Medical therapies such as pirfenidone and nintedanib have been approved for PF treatment, but novel therapies continue to be researched due to limited efficacy and unwanted side effects. Such novel therapies include romidepsin that manipulate genetic acetylation to reduce fibrosis-promoting pathways [5,6].

In PF caused by unilateral absence of a pulmonary artery, the disease process can often go undetected since the other functioning lung provides adequate oxygenation [8]. However, in patients with symptoms such as hemoptysis and worsening dyspnea on exertion, treatment may be necessary to avoid long-term complications such as blood loss and pulmonary hypertension. Treatment options in these patients include embolization of collateral pulmonary vasculature and/or pneumonectomy [8,9].

## Case presentation

This case involves a 30-year-old female with presenting symptom of worsening shortness of breath. She had a remote history of mild asthma during childhood but suffered no long-term sequelae. She was never a smoker.

**Figure 1:**
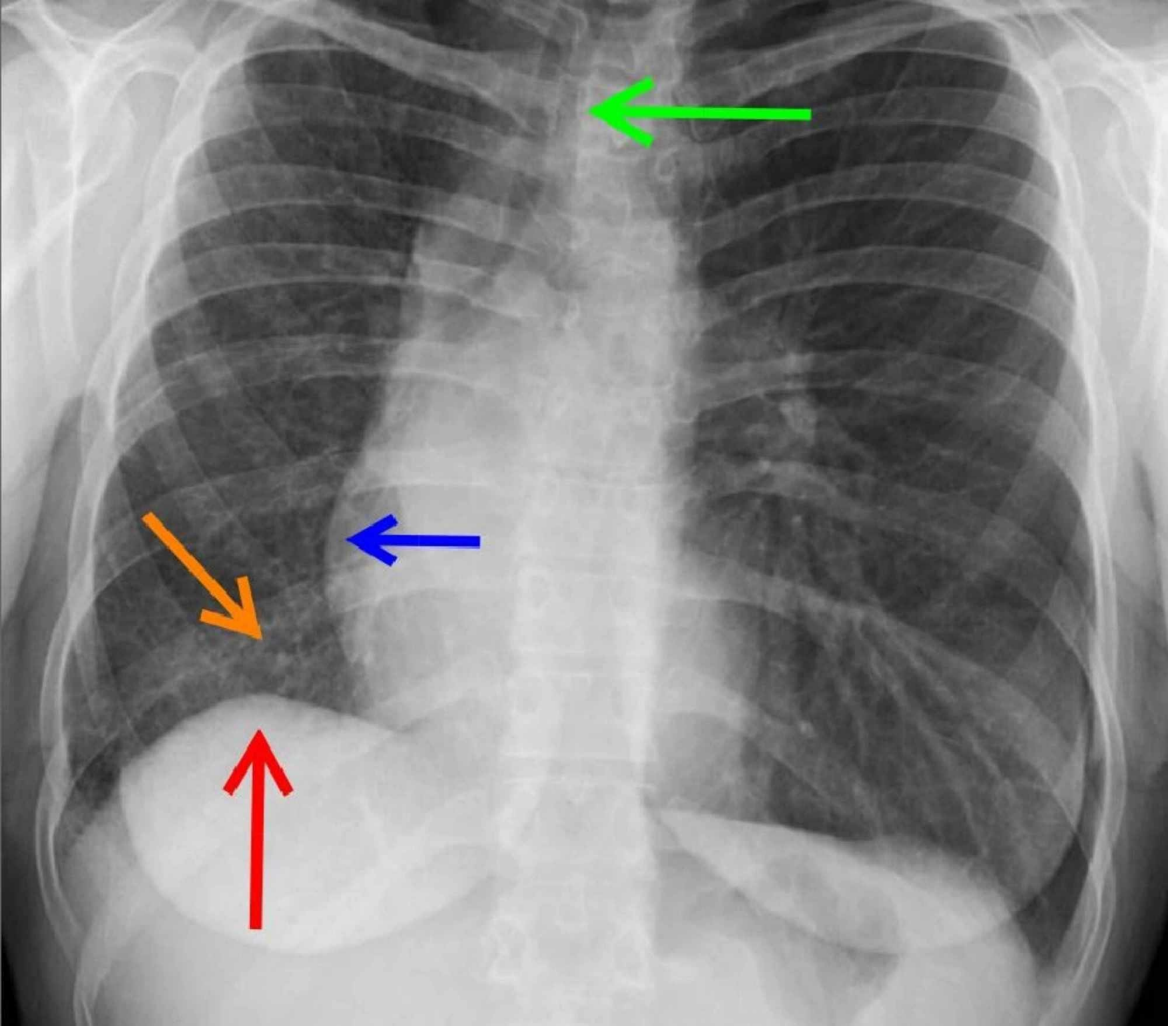
Chest X-ray evaluating causes for dyspnea. The radiograph shows right lung volume reduction manifested by tracheal deviation (green arrow), mediastinal shift (blue arrow), and elevation of the right hemidiaphragm (red arrow). There is also increased prominence of interstitial markings and reticulation of the right lung (orange arrow). The left lung shows no disease on plain film.

Clinical workup including history, physical exam, electrocardiogram (EKG), and lab testing was not revealing for any inciting factor of dyspnea until a chest X-ray was completed which showed elevated right hemidiaphragm, tracheal deviation, and mediastinal shift (Figure 1).

**Figure 2:**
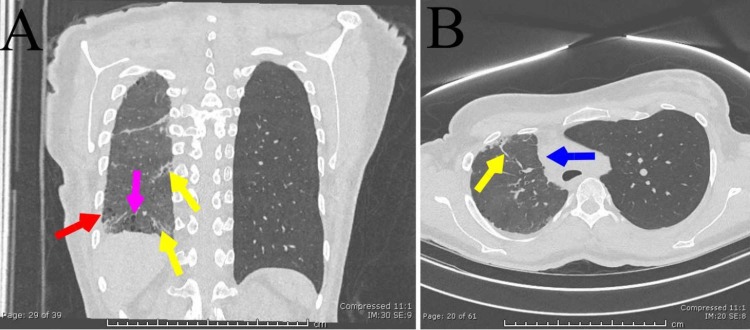
Fibrotic lung disease. Coronal (A) and axial (B) non-contrast computerized tomography images show fibrotic septal thickening (yellow arrows) and honeycombing (red arrow) with bronchiectasis (pink arrow). The mediastinum is displaced to the right (blue arrow).

**Figure 3:**
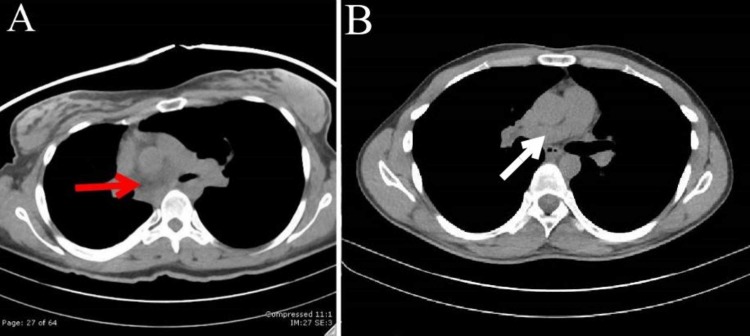
Absence of the right pulmonary artery. Non-contrast computerized tomography axial image of the patient (A) and a person with normal vasculature (B) at the level of the pulmonary arteries. The white arrow indicates normal right pulmonary artery appearance while the red arrow depicts absence of the right pulmonary artery in the region posterior to the ascending aorta where it typically resides.

Follow-up computerized tomography (CT) study revealed fibrotic right lung parenchyma (Figure 2) and the absence of the right pulmonary artery (Figure 3).

Upon further outpatient testing, and noting the absence of hemoptysis and severe shortness of breath (SOB), the patient and her clinician agreed upon conservative management.

## Discussion

With risk factors such as advanced age, smoking, and environmental exposure being absent, the congenital absence of the right pulmonary artery is likely the causative agent for the patient’s unilateral pulmonary fibrosis. This is further supported by the fact that the patient’s other lung is healthy though the disease is usually bilateral.

Pulmonary fibrosis in patients with congenital absence of a pulmonary artery has been attributed to hypoperfusion [8]. Fibrosis of the right lung may have been due to increased dose of ROS in this patient. Without the supply of deoxygenated blood from the right pulmonary artery to provide a gradient for oxygen to diffuse away from the aerated lung parenchyma, the previously-functioning tissue likely suffered from increased oxidative stress causing the eventual unilateral pulmonary fibrosis [7]. Although there is no consensus treatment for pulmonary fibrosis related to unilateral absence of a pulmonary artery, anti-fibrotic therapies that are usually the mainstay of treatment for idiopathic PF can be speculated to help pulmonary fibroses due to other causes. If anti-oxidant drugs can be targeted to act at the pulmonary parenchyma, they may prove useful too, since these patients have exacerbated exposure to ROS which are shown to increase the activity of TGF-β, a key player in PF manifestation [7].

In later stages of PF caused by unilateral absence of a pulmonary artery, however, treatments may require more invasive therapies such as ipsilateral embolization of collateral pulmonary vasculature and/or pneumonectomy [8,9].

## Conclusions

This 30-year-old female patient with no known respiratory risk factors is an unlikely candidate for the development of pulmonary fibrosis. The presence of her unilateral pulmonary fibrosis and ipsilateral absence of right pulmonary artery provide a unique mechanism for her disease pathophysiology which may include increased reactive oxygen species exposure with TGF-β as a mediator. This may suggest unique therapies to treat the disease process.
